# The Microscopic Anatomy of the Human Body in Health and Diseases, Illustrated with Numerous Drawings in Colour

**Published:** 1846-10

**Authors:** 


Art. II.—*The Microscopic Anatomy of the Human Body in Health and Disease, illustrated with numerous Drawings in Colour.* By Arthur Hill Hassall, f.l.s., m.r.c.s., &c.—Parts I and II. 8vo, pp. 76. With six Plates.

The design of this work is very commendable. The present rapid advance of microscopic inquiry, together with the increasing importance of its results, fairly demand that a special treatise should be devoted to it; and a volume so moderate in size and price as this promises to be, would deserve every encouragement if its performance were equally satisfactory. This, however, we regret to say, is not in our opinion the case, in regard to any one of the points on which the author claims a favorable consideration. After stating the intended number of parts to be twelve, he proceeds—"It is intended to embrace a systematic and copious *illustrated* description of the various fluids and solids of the human frame, no structure or organ of the body being omitted." And he afterwards adds, "The design of the work, if not altogether original, is almost unique; the only work extant which embraces the entire range of microscopic anatomy, and this *not exclusively human,* is that of Mandl." Why, Mr. Hassall's work is not a whit more exclusively human than that of Mandl. Of the *three* plates in the first number, only *one*—and of the *three* in the second number, only *half a one*—represents objects of human anatomy. We should not think the worse of the book on this account, if it were not that if the other subjects are treated upon the same scale with the blood and circulation (which occupy the two parts already published), the book must extend to fifty parts instead of twelve; and that the fact is plainly inconsistent with the author's preliminary announcement. Further, Mr. Hassall says, "One great feature of the book, it is hoped, will be the *fidelity of the drawings;* a principal fault in most of the published works on minute and microscopic anatomy being that the figures do not exhibit the characters of the text." Here, again, we feel obliged to state that Mr. Hassall's figures are *very* far from bearing a resemblance to nature, either in their drawing or colouring. The drawing is in coarse lithography, which is not at all calculated to give an idea of the real aspects of the objects. And the colouring will strike every practised microscopist, we venture to affirm, as egregiously incorrect, and calculated to mislead. In all the figures of red corpuscles of the blood, for example, these objects are represented as of a full red tint, even when lying in a single layer; when every tyro knows that the hue is scarcely distinguishable in a single disc, and that the red hue becomes apparent only when two or three overlap. The differences between the red and colourless corpuscles of the human blood, in the very first figure, are thus grossly exaggerated; as is manifest from the fact, that no one could overlook them in the plate, whilst in nature they have only been lately detected by a careful scrutiny. We recommend to Mr. Hassall's attention the admirable figures of Donné, which are engravings of high finish and great beauty, faithfully copied from daguerreotype images of the objects themselves; as showing what blood-corpuscles really resemble under the microscope.

We greatly regret that we are obliged to speak thus disparagingly of Mr. Hassall's very laudable attempt. But our duty to the medical public requires, that as the subject is one on which, from the attention we have given to it from the very first, we feel well qualified to form an opinion, this opinion should be expressed as unreservedly as if it were more favorable. Should future numbers present such an improvement in their character, as to warrant our commendations instead of our condemnation, we shall most gladly bestow them.

